# Transition of adult patients with pediatric orthostatic intolerance from child-centered care to adult-centered care

**DOI:** 10.3389/fped.2022.946306

**Published:** 2022-10-28

**Authors:** Yoshitoki Yanagimoto

**Affiliations:** ^1^Department of Pediatrics, Kansai Medical University, Hirakata, Japan; ^2^Department of Pediatrics, Kansai Medical University Medical Center, Moriguchi, Japan

**Keywords:** orthostatic intolerance (OI), cardiovascular deconditioning, psychiatric disorders as a secondary problems, transition, collaboration system between pediatric and adult care

## Introduction

Orthostatic intolerance (OI) is defined by difficulty tolerating upright posture because of symptoms that abate when returned to a supine position (1). Essential symptoms of OI include lightheadedness, headache, fatigue, weakness, nausea, exercise intolerance, tachycardia, and hypotension (1, 2). OI is caused by autonomic nervous system dysfunction that occurs most often in adolescence. Although symptoms resolve by adulthood in most cases, decreased quality of life has been reported to persist into adulthood for some patients, both in Japan and internationally (1–3). Therefore, the treatment of OI that persists into young adulthood has become an important issue in the field of psychosomatic and behavioral pediatrics (4).

Few studies have examined the transition of adult patients with OI from pediatric to adult medical care. OI has traditionally been considered to be a transient condition that is restricted to children and adolescents. However, recent evidence suggests that OI can present as a major disabling illness in teenagers and young adults, and reduce their quality of life (2). In Japan, OI is generally known as orthostatic dysregulation (OD). Although the Japanese Society of Psychosomatic Pediatrics previously reported that 90% of OD patients improve by age 18 (5), they recently reported that an increasing number of patients exhibit symptoms that persist into adulthood (6). The reason for this phenomenon has not yet been clarified, but may be related to both physical deconditioning caused by changes in the lifestyles of young people, and psychiatric comorbidities such as anxiety, and depression caused by low self-esteem resulting from maladaptation to social activities. To improve the prognosis of adult patients with child-onset OI, it is necessary to develop an approach to transition from child-centered to adult-centered care that focuses primarily on patients' independence.

## Overview of pediatric orthostatic intolerance

### Clinical features

OI is a common disease in teenagers caused by a disorder of the autonomic nervous system that impairs circulatory control during orthostasis, resulting in decreased cerebral blood flow and a range of symptoms: general malaise, headache, dizziness, nausea, and difficulty waking (1). Typical features of OI in childhood and adolescence are divided into three types: orthostatic hypotension, postural tachycardia syndrome, and vasovagal syncope (1, 7). It has been reported that most cases of OI improve between adolescence and adulthood (7), and that symptoms may remain but are typically not problematic for daily living and do not require treatment (8). However, deconditioning and secondary psychiatric disorders have prolonged OI symptoms, increasing the number of adults with OI (1, 9).

### Cardiovascular deconditioning

Cardiovascular deconditioning has been reported to be one of the causes of OI (10). Resulting from myocardial atrophy, cardiovascular deconditioning involves decreased cardiac output, decreased circulating plasma volume, and muscle atrophy in the lower body caused by the microgravity environment, outer space, and reduced physical activity, resulting in circulatory ataxia during standing and reduced orthostatic tolerance (11, 12). Additionally, deconditioning can occur when physical activity in daily life is reduced because of the symptoms caused by OI, and these symptoms may be further aggravated as a result. Deconditioning was reported to occur in healthy children during lockdown in response to coronavirus disease 2019 (13). Thus, prolonged OI caused by deconditioning may be an increasingly important issue because of the lifestyle changes caused by responses to the coronavirus disease 2019 pandemic.

Few pediatricians provide appropriate medical care to patients with prolonged OI, although it is common for physicians to treat patients with deconditioning such as sarcopenia or disuse syndrome (14, 15). It may be possible to improve prolonged OI by improving deconditioning through collaboration with specialists treating disuse syndrome or sarcopenia.

### Psychiatric disorders as a secondary problem

The persistence of OI symptoms over time and the associated limitations on social activities can reduce patients' self-esteem and quality of life. This can result in psychiatric complications such as anxiety disorders and depression (16, 17). In patients with prolonged OI, it is difficult to determine whether physical symptoms are caused by OI, anxiety, or depression. If OI is considered to be an autonomic imbalance associated with growth, it may be reasonable to assume that the physical symptoms in patients approaching adulthood are caused by an anxiety disorder. Patients with psychiatric complications often have difficulty engaging in social activities, including difficulty going out, because of long-term social withdrawal and symptoms such as anxiety. Social withdrawal can lead to a lack of exercise, exacerbating deconditioning, prolonging OI symptoms, and resulting in a vicious cycle.

## Discussion

### Needs and obstacles involved in the health care transition of adult patients with pediatric OI

OI has traditionally been considered as a disease with a high prevalence in childhood and adolescence, with symptoms of OI improving as children grow up. However, a recent study in Japan reported that symptoms of OI have become increasingly prolonged and more likely to persist into adulthood (6) because of complications related to cardiovascular deconditioning and psychiatric disorders as secondary problems. Thus, it is necessary to establish a transition system in OI. However, adult patients with childhood-onset OI may face difficulty adapting to adult medical care because of their immaturity, lack of independence, and lack of social experience. Lack of independence among patients has been reported in cases of transition in other chronic pediatric diseases (18–22), and this is also the case for patients with OI. To improve the prognosis of adult patients with prolonged OI symptoms, an approach toward healthcare transition that focuses primarily on patients' independence may be effective.

The lack of knowledge and experience, and the poor coordination of care between pediatricians and adult care providers can also hinder transitions (21, 23, 24). Adult health care providers are often unfamiliar with the care of complex pediatric patients who are approaching adulthood, and may therefore be uncomfortable managing them (23). Pediatricians should support patients' transition by providing consultation from the adult department during their move and afterwards, as required. Additionally, daily collaboration with acceptable transition sites in the community is considered to be necessary to improve the second concern (6, 25).

### Proposal regarding the process of transition

There is no generally accepted transition system in OI. Therefore, transition models in other chronic diseases should be used as a guide for OI. As a concrete way to start the transition process, the first step is to determine the age of transition. The recommended age for beginning the transition has been proposed to be around the 13th birthday (25), or 14–18 years old (26). In my opinion, the age of 15 years is an appropriate point at which to discuss transition with patients and their parents, because compulsory education in Japan ends at 15 years of age, and parents and their patients are likely to be aware of the termination of pediatric care. In addition, this is an appropriate time to begin preparing patients and their parents for graduation from pediatrics. Around the end of the patient's compulsory education, I always give guidance about the patient graduating from pediatric practice. I suggest that the patient's own choice of treatment is an essential issue regarding independence. The patient's own decision-making, rather than that of their guardians, regarding the proposed treatment plan is important for promoting the patient's independence from their guardians. Patients' experience making their own decisions may improve their self-esteem. This process not only facilitates independence but may also help to prevent secondary psychiatric disorders. In the process of the patient becoming more independent, I recommend using the Transition Readiness Assessment Questionnaire (TRAQ) for assessment of self-management and self-advocacy as a marker for transition readiness (27). I believe that the use of TRAQ enables the health care provider to confirm the progress of transition readiness in adult patients with prolonged OI, as in other types of childhood-onset chronic illness.

The next important step is to choose a model of transition. In OI, a transition system has not yet been established, in Japan and internationally. Several transition programs and guidelines for other chronic diseases are available. I recommend the three types of transition models by Angela et al. (28): “transfer with referral letter,” “joint clinic,” and the “teenager/transition clinic” mode. The model that best fits regional context should be chosen. Transfer with referral letter is the most convenient method, but transitions may occur without communication between pediatricians and adult physicians. The joint clinic type is based on collaboration between medical institutions. This approach is highly effective for reducing patient anxiety but takes time and financial resources. According to the “teenager/transition clinic” model, Crowley et al. reported that this is one of the most commonly used strategies in successful transition programs (29). I believe that the use of remote interviewing could reduce burdens, and may be helpful in all models. In addition, appropriate transition sites should be considered as adult departments that can treat deconditioning and secondary psychiatric disorders.

### Destination of transition

When deciding on which adult department to transfer the patient to, the most appropriate destination differs depending on whether the main problem is physical symptoms caused by deconditioning or secondary psychiatric disorders. Considering the pathophysiology, the department of cardiology or rehabilitation that created the structured exercise training and rehabilitation plan should be selected as a counterpart for cases with prolonged OI mainly caused by cardiovascular deconditioning. For cases in which secondary psychiatric disorders are the main problem, psychiatrists are the preferred transition destination. In some cases, the patient will need to transition to more than one medical facility. The pediatrician should serve as a link between these multiple medical providers. Although the most appropriate model in OI depends on the local health care system, I believe the “joint clinic” model is most appropriate in OI for the reasons described above.

Finally, I would like to stress the importance of the attitude of the pediatrician. Pediatricians' attitude toward adult patients with OI who have completed the transition should be similar to the attitude of teachers toward students that have graduated. Pediatricians should be available to meet with the patient even if they are no longer directly involved in the consultation, and to congratulate the patient on their progress to adult care. Pediatricians should continue to watch over their patients, similar to the teachers of students who have graduated.

## Author contributions

YY contributed to conception, design of the study, and wrote the first draft of the manuscript.

## Conflict of interest

The author declares that the research was conducted in the absence of any commercial or financial relationships that could be construed as a potential conflict of interest.

## Publisher's note

All claims expressed in this article are solely those of the authors and do not necessarily represent those of their affiliated organizations, or those of the publisher, the editors and the reviewers. Any product that may be evaluated in this article, or claim that may be made by its manufacturer, is not guaranteed or endorsed by the publisher.
